# Music, reward and frontotemporal dementia

**DOI:** 10.1093/brain/awu145

**Published:** 2014-06-11

**Authors:** Phillip D. Fletcher, Camilla N. Clark, Jason D. Warren

**Affiliations:** Dementia Research Centre, UCL Institute of Neurology, University College London, London, UK

Sir, The recent study by Perry *et al.* (2014) draws attention to the important issue of abnormally enhanced reward-seeking by patients with frontotemporal dementia (FTD). This issue presents major challenges for the clinical management of these patients and provides a unique window on the neurobiology of brain network disintegration in a diverse group of neurodegenerative pathologies. While Perry and colleagues emphasize seeking of stimuli with clear biological reward potential (sweet foods, drugs of abuse and sex), abnormal reward-seeking in FTD is not restricted to such stimuli. Indeed, one of the most potent inducers of such behaviour in patients with FTD is a stimulus with no clear biological value: music. Abnormal, intense craving for music (musicophilia) is common in FTD and has a cerebral correlate centred on the mesial temporal lobe (Fletcher *et al.*, 2013).

Both in functional imaging studies of the healthy brain and in neurodegenerative disease (Omar *et al.*, 2011; Salimpoor *et al.*, 2013; Clark *et al.*, 2014), music has been shown to engage striatal and basal forebrain regions overlapping or intimately connected with those demonstrated by Perry *et al.* (2014), in addition to a distributed network of cortical and limbic structures. Why music should behave as a biologically rewarding stimulus remains unresolved although clues may lie with its capacity to encode emotional mental states (Clark *et al.*, 2014): a cognitive process that is also often catastrophically disrupted in FTD.

We therefore suggest music as a promising candidate model system for addressing some of the key questions for future work raised by the study of Perry and colleagues. In particular, as a universal and widely valued, yet abstract stimulus, music is ideally suited to probe interactions between reward, affective and cortical information processing circuitry (Omar *et al.*, 2011; Salimpoor *et al.* 2013). This circuitry is inherently vulnerable in FTD. Moreover, a stimulus that can dissect apart the components of such large-scale brain networks may enable hedonic and physiological phenotyping of a range of other neurodegenerative disorders (including Parkinson’s disease) that also cause profound derangements of reward processing.
